# The influence of demographic, social-educational determinants and diabetes management on agreement between glucometer and logbook and its impact on glycemic control in patients with type 1 diabetes: a follow-up study

**DOI:** 10.1186/s13098-019-0443-9

**Published:** 2019-06-17

**Authors:** Rebeca Cavalcante, Alessandra S. M. Matheus, Aneliza Zanette, Bruna Braga, Bruna Duarte, Bruna Würdig, Daniele Maieron, João Scarparo Sorio, Luciana Bagatini, Michelle Cherit, Marilia Brito Gomes

**Affiliations:** 1 Department of Internal Medicine, Diabetes Unit, State University Hospital of Rio de Janeiro, Rio de Janeiro, RJ Brazil; 2 Manaus, Brazil

**Keywords:** Type 1 diabetes, Glycemic control, Socio-educational determinants, Self-monitoring blood glucose, Glucometer

## Abstract

**Background:**

The primary objective of this study was to evaluate the demographic, clinical, social-educational determinants and diabetes management factors that have influenced the agreement between glycaemia obtained from a glucometer and logbook; the second objective was to evaluate the influence of the above-mentioned factors on glycemic control and its trajectories in Type 1 diabetes (T1D) over 1 year follow-up period during routine clinical practice.

**Methods:**

This was a prospective observational cohort study conducted at the Diabetes Unit at Rio de Janeiro’s State University, between May 2017 and May 2018. All consecutive patients with clinical diagnosis of T1D that attended the Diabetes Unit between April and June 2017 were enrolled in this study.

**Results:**

Data were obtained from 158 patients. Overall, for 112 (73.2%) of the patients, we found no agreement between glycaemia obtained from a glucometer and the logbook (group 2). In 41 (26.8%) of the patients there was an agreement (group 1). Patients from group 1 presented a lower mean glycated hemoglobin (HbA1c) (p = 0.03) and a tendency to have a lower baseline HbA1c (p = 0.08), they received more frequently strips for glucose monitoring from the Sistema Único de Saúde (SUS) (p = 0.047) and were more adherent to the prescribed diet (p = 0.01) than patients from group 2. Multivariate analysis of this agreement (as a dependent variable) showed that adherence to diet was the only significant independent variable. Significant difference was noted between baseline and final HbA1c [(9.4 ± 2.2%) vs (9.03 ± 1.8%), p = 0.017], respectively.

**Conclusions:**

Our study revealed that the majority of T1D patients that were followed at a tertiary center did not have significant agreement between glycaemia obtained from a glucometer and a logbook. Adherence to diet was the main factor related to the agreement, but the supply of strips by SUS should also be considered in clinical practice.

## Background

Type 1 diabetes (T1D) is a chronic disease with an incidence rate that is increasing worldwide [1, 2]. Treatment includes intensive insulin therapy and non-pharmacological therapies, both important tools for obtaining an adequate glycemic control that is necessary to avoid or postpone diabetes-related chronic complications as has been demonstrated by the Diabetes Control and Complications Trial (DCCT) and the Epidemiology of Diabetes Interventions and Complications (EDIC) study [3, 4]. Nevertheless, in routine clinical practice, attaining adequate glycemic control remains a challenge mainly in developing countries and among minorities [5, 6]. A recent multicenter study conducted in the United States of America (USA), the Type 1 Diabetes Exchange study, showed that the majority of T1D patients across all age-groups did not meet the American Diabetes Association’s (ADA) target for good glycemic control and showed an overall mean glycated hemoglobin (HbA1c) of 8.4% (68 mmol/mol) [7]. In this study, the majority of patients was under continuous subcutaneous insulin infusion (CSII) therapy and performed self-monitoring of blood glucose (SMBG) 4–6 times per day, which is currently an important tool for diabetes care that helps patients, caregivers and the diabetes team to adjust insulin therapy during hypoglycemia, hyperglycemia, physical activity, stress, illness and carbohydrate counting [8]. Despite the aforementioned advantages, there are many barriers for the effective implementation of SMBG into routine clinical care that include poor education, fear of finger prick-related pain and difficulty in using the results to adjust insulin dosage [9]. In developing countries, the cost of the strips and the compliance of the patients in bringing the glucometer to routine clinical visits, an important component for comparing data with the logbook, could also be significant barriers [9]. Currently, many guidelines for SMBG exist worldwide, such as those from ADA and the Brazilian Diabetes Society (SBD) [10, 11], which recommend at least three SMBG daily in patients with T1D. However, there is a considerable gap between the recommended and the reported frequencies of SMBG in T1D patients [12]. In general, there is a high degree of variability in the daily frequency of SMBG between patients, which range between one and five or more times daily [7, 12]. The effect(s) of the frequency of SMBG on glycemic control is controversial because although most studies show improvements in glycemic control with higher SMBG frequencies [12, 13], in most of them the patients were still out of goal for good glycemic control. Considering the aforementioned study results, diabetes has become a major health issue due to its high related costs, both direct and indirect, for the health care systems [14, 15]. The primary objective of the present study is to evaluate the demographic, clinical, social-educational determinants (SED) and diabetes management factors that influence the agreement between glycaemia obtained using a glucometer, and the logbook, the second one is to evaluate the impact of this agreement upon glycemic control. This study also evaluates the influence of the above-mentioned factors on glycemic control and its trajectories for patients with T1D throughout 1 year of follow-up during routine clinical practice.

## Methods

This was a prospective observational cohort study conducted at the Diabetes Unit from Rio de Janeiro’s State University (UERJ) between May, 2017 and May, 2018. All consecutive patients with T1D attending the Diabetes Unit from May to June of 2017 were enrolled in the study. Included patients must have had at least two clinical visits during this period. All patients received health care from Sistema Único de Saúde (SUS), which guarantees free healthcare for every Brazilian citizen and also free neutral protamine hagedorn (NPH) insulin and regular insulin, syringes, needles, glucometers and strips for blood glucose monitoring. T1D diagnosis was made by a physician if the patient had a typical clinical presentation of T1D that included variable degrees of hyperglycemia, weight loss, polyuria, polydipsia, polyphagia and the need for continuous insulin use since diagnosis. Patients younger than 13 years of age were categorized as children, patients between 13 and 19 years old were categorized as adolescents, and patients older than 19 as adults [16]. This study was approved by the ethics committee of Pedro Ernesto University Hospital (HUPE) of the UERJ. The following variables were assessed using a questionnaire or medical records: current age, age at diagnosis, diabetes duration, self-reported color-race, height (m), weight (kg), insulin dose (U/kg), frequency of SMBG and self-reported adherence to diet (determining if the patient followed the prescribed diet at least 80% of the time) [17]. Factors that are related to diabetes management included bringing the logbook and the glycaemia obtained from a glucometer, i.e., blood glucose was checked directly on the glucometer during every medical appointment, if the patients lend their glucometer to other people, besides the acquisition of supplies (glucometers and strips for blood glucose monitoring) through the SUS, were also evaluated. Body mass index (BMI) was determined by dividing the individual’s weight (kg) by the square of the individual’s height (m^2^). As part of our routine clinical care, patients were advised to bring the glucometer and logbook to each clinical visit.

The percentage of agreement between the glycemic levels obtained from the glucometer and from the logbook, i.e., glycaemia values recorded in the logbook compatible with those directly checked in the glucometer, was calculated and considered as follows: a positive agreement was determined if there was at least 90% of agreement between both methods during all clinical visits over 1 year (group 1); a category of no agreement was determined if the percentage of agreement was lower than 90% (group 2) [18]. The present study adopted the following ADA goals for adequate metabolic and clinical control [19]: good glycemic control (HbA1c at goal) was defined as an HbA1c < 58 mmol/mol (7.5%) for T1D patients up to 19 years old and < 53 mmol/mol (7%) for adult T1D patients and poor glycemic control was defined as an HbA1c of ≥ 75 mmol/mol (9%). HbA1c was measured using high-performance liquid chromatography (HPLC, Bio-Rad Laboratories, Hercules, California, USA). Economic status was defined using the Brazilian Economic Classification Criteria, [20] and the following classes of economic status were considered for this analysis: high, middle, low and very low.

### Statistical analysis

First, exploratory data analysis was performed and these data are presented as mean ± standard deviation (± SD) or median, interquartile range [IQR] for continuous variables and numbers (relative frequencies) for discrete variables. Comparisons between independent continuous variables were performed using T tests, Mann–Whitney or Kruskal–Wallis, as indicated. Comparisons between continuous dependent variables were performed using paired T tests or Wilcoxon rank tests when indicated. Pearson’s Chi squared or Fisher tests were used to compare discrete variables. We performed a multivariate logistic regression with agreements that were measured between the glycaemia values obtained from the glucometer and logbook (yes or no) for all clinical visits and was treated as the dependent variable and self-reported color-race (Caucasian or non-Caucasian), economic status, age, adherence to diet, duration of diabetes, and supply of blood glucose test strips by SUS were treated as independent variables. Multivariate stepwise linear analysis was performed using the final levels of HbA1c as well as with the mean values of HbA1c during the 1 year follow-up as the dependent variable. Variables with a Pearson’s correlation *p* value < 0.2 such as economic status, age, adherence to diet, duration of diabetes, supply of blood glucose test strips by SUS, time of follow-up, years of study and living in the city of Rio de Janeiro were considered independent variables in the above multivariate model. As a second step, a multivariate stepwise linear hierarchical analysis was run to evaluate if there were statistically significant increases in *R*^*2*^ after the addition of each independent variable. The Nagelkerke R-squared value was also calculated. Analyses were performed using Statistical Package for the Social Sciences version 17.0 (SPSS, Inc., Chicago, Illinois). Odds ratios with 95% confidence intervals (CIs) were expressed as indicated. A two-sided *p* value less than 0.05 was considered significant.

## Results

### Overview of the demographic, clinical and laboratory baseline data of the studied population

The demographic, clinical and laboratory data are described in Table 1.

**Table 1:** Baseline demographic, clinical and laboratory data of the studied population

Variable	Value
N	158
Female, n (%)	79 (50.0)
Age, year^a^	22 [19]
Duration of diabetes, year	9.0 [14.0]
Age at diagnosis, year	13.0 [10.0]
Time of follow-up, year	9.0 [15]
Years of study	10.0 [5.0]
Self-reported color-race, n (%)	
Caucasian	84 (53.2)
Non-Caucasian^b^	74 (46.8)
Economic status, n (%)	
High	0
Medium	31 (19.6)
Low	114 (72.2)
Very low	13 (8.2)
HbA1c (%)	9.0 [2.95]
HbA1c (mmol/mol)	75.41 [32.24]
Glucometer, yes n (%)^c^	87 (55.1)
Log-book, yes n (%)^c^	122 (77.2)
SMBG, yes n (%)	134 (84.8)
SMBG, n	3.0 [1.0]
Insulin dose, U/kg	0.8 ± 0.4
BMI	23.4 ± 4.9
Adherence to diet, yes n (%)	35 (22.2)

Data are presented as number (percentage), median [interquartile range, IQR] or mean ± SD

*Y * year,*SMBG* self-monitoring blood glucose

^a^20 children (12.7%), 39 adolescents (24.7%), 99 adults (62.7%)

^b^African-Brazilians, Mulattos, Asians, and Native Indians

^c^Number of patients who brought the glucometer and the logbook to the clinical appointment

Overall, 158 patients were included in the baseline study; 126 (79.7%) patients lived in the city of Rio de Janeiro; 59 (37.4%) were under 19 years old and 99 were (62.7%) adults. According to glycemic control baseline data, 25 patients (15.9%) were within the target for good glycemic control, that is, < 7.0% for adults and < 7.5% for patients under 19 years, and 94 patients (53.8%) were considered to have poor glycemic control (HbA1c ≥ 9.0%) and 48 (30.4%) patients were within intermediate HbA1c (7.1–8.9% for adults and 7.5–8.9% for children and teenagers). At the end of the study, the total of good (HbA1c < 7% and < 7.5%, for adults and patients under 19 years old, respectively) and poor glycemic control (HbA1c > 9%) patients were 13 (8.2%) and 64 (40.6%), respectively, and at the intermediate status there were 76 patients (48.6%). Data according to the stratification of baseline and final HbA1c are described in Table 2.

**Table 2:** Total of patients stratified according to HbA1c levels and age at baseline and at the final visit

A1c	Baseline		Final	
	< 19 year	≥ 19 year	< 19 year	≥ 19 year
	N = 59	N = 99	N = 56	N = 97
1. < 7.0%		21 (13.3)		9.0 (5.9)
< 7.5%	4.0 (2.6)		4.0 (2.7)	
2. ≥ 7.0–8.9%		36 (22.8)		50 (32.7)
≥ 7.5–8.9%	12 (7.6)		26 (17)	
3. 9–9.9%	12 (7.6)	21 (13.3)	6 (3.9)	20 (13.1)
4. ≥ 10%	31 (19.6)	21 (13.3)	20 (13.1)	18 (11.8)

Y = years old; data are presented as number (percentage)

* p = 0.004 for comparison between groups

** p = 0.13 for comparison between groups

### Overview of the demographic, clinical and laboratory data from the studied population according to the agreement between glycaemia obtained from glucometer and logbook records during the study

**Fig. 1:**
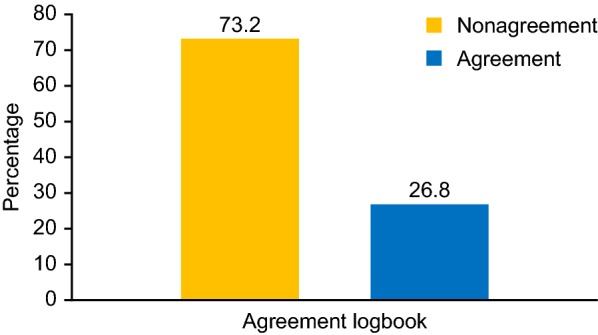
Percent of patients that had agreement between the glucometer and the logbook records

Considering the pooled group, five patients were excluded because they only had one appointment during the follow up period, which resulted in a sample of 153 patients (96.8%). After the first visit, patients were advised to bring the glucometer and logbook. 112 (70.5%) patients had no agreement between the glycaemia obtained from the glucometer and the logbook records (group 2). In 41 (29.5%) of these patients, there was agreement (group 1), as shown in Fig. 1. Patients from group 1 had lower mean HbA1c values (p = 0.01), a tendency for a lower baseline HbA1c (p = 0.08) and lower final HbA1c (p = 0.1) values than patients from group 2. These patients received strips for glucose monitoring from the SUS more frequently (p = 0.04) and were more adherent to the prescribed diet (p = 0.01) than patients from group 2. These data are described in Table 3. A multivariate analysis was performed to evaluate the effects of self-reported color/race (Caucasian or non-Caucasian), economic status, age, adherence to diet, number of daily SMBG and supply of blood glucose test strips by the SUS on the likelihood that patients have an agreement between glycaemia obtained from the glucometer and the logbook. The adjusted model revealed that the independent variable can explain 8.2% (Nagelkerke R-squared) of the variance for a given patient to have agreement between the glycaemia obtained from the glucometer and the logbook. After adjustment, the significant independent variable that was associated with having agreement was the patients’ adherence to diet [B = 1.038, OR = 2.728; 95% CI (1.205–6.173); p = 0.04].

**Table 3:** Clinical, demographic and laboratory data according to the agreement between glycemia obtained from glucometer and the logbook records

Variable	Group 1	Group 2	p-value
N (%)	41 (26.8)	112 (73.2)	
Gender, female n (%)	25 (61.0)	52 (46.4)	0.3
Duration of diabetes (year), median [IQR]	8.0 [17.0]	10.0 [13.0]	0.4
Age (year), mean ± SD	27.7 ± 15.2	26.3 ± 15.7	0.6
Mean age at diagnosis (year), mean ± SD	15.1 ± 10.6	15.2 ± 9.9	0.9
Age range, n (%)			
1–13 year	9 (22.0)	9 (8.0)	
14–19 year	8 (19.6)	30 (26.8)	0.055
> 19 year	24 (58.6)	73 (65.2)	
Years of study (year), mean ± SD^a^	9.6 ± 4.8	10.3 ± 3.6	0.3
Time of follow-up (year), median [IQR]	8 [17.0]	9 [13.7]	0.3
Ethnicity, n (%)			
Caucasian	24 (58.5)	57 (50.9)	0.2
Economic status, n (%)			0.6
High	0	0	
Medium	10 (24.4)	20 (17.9)	
Low	27 (65.9)	83 (74.1)	
Very low	4 (9.8)	9 (8.0)	
Living in Rio de Janeiro City, yes n (%)	31 (75.6)	90 (80.4)	0.5
Insulin U/kg	0.8 ± 0.4	0.8 ± 0.2	0.8
Glycemic control			
HbA1c (%), baseline	8.9 ± 1.8	9.6 ± 2.3	0.08
HbA1c (mmol/mol), baseline	82.1 ± 25.9	74.6 ± 20.8	
HbA1c (%), final	8.6 ± 1.6	9.1 ± 1.9	0.1
HbA1c (mmol/mol), final	76.9 ± 21.3	70.9 ± 17.6	
HbA1c (%), mean in 1 year	8.7 ± 1.3	9.4 ± 1.8	0.01
HbA1c (mmol/mol), mean in 1 year	79.8 ± 19.5	71.7 ± 14.2	
HbA1c (good), baseline; n (%)^b^	7 (17.0)	18 (16.0)	0.5
HbA1c baseline ≥ 9.0%; n (%)^c^	18 (43.9)	62 (55.5)	0.4
Hb1Ac (good), final; (%)^b^	3 (7.3)	10 (8.9)	0.2
HbA1c final ≥ 9.0%; n (%)^c^	12 (29.2)	52 (46.4)	0.09
HbA1c (good) (mean), (%)^b^	3 (7.3)	6 (5.4)	0.3
HbA1c mean, ≥ 9.0%; n (%)^c^	15 (36.5)	54 (48.2)	0.2
Diabetes management			
SMBG, n	3.4 ± 1.1	3.2 ± 1.2	0.3
Supply of glucose strips from SUS, n (%)	33 (80.5)	70 (62.5)	0.04
Adherence to diet, yes n (%)	15 (36.6)	19 (17.0)	0.01
BMI (kg/m^2^)	22.5 ± 4.5	23.7 ± 4.8	0.2

The data are presented as a percentage, mean ± SD or median [IQR]

* y*  year,* F* female, *SMBG* self-monitoring of blood glucose, *SUS* Sistema Único de Saúde

^a^Years of study was considered only in adult patients

^b^HbA1c at goal was defined as HbA1c < 7.5% (58 mmol/mol) for T1D patients between 13 and 19 years old and < 7.0% (53 mmol/mol) for adult T1D patients; ^c^HbA1c > 9.0% (75 mmol/mol) was defined as poor glycemic control

### Overview of the demographic, clinical and laboratory data of the studied population, agreement between glycaemia obtained from glucometer and logbook records during the study and glycemic control

The final sample considered for this analysis was in regards to 153 (96.8%) patients. A significant difference was found between baseline HbA1c and final HbA1c values [(9.44 ± 2.2%) vs (9.03 ± 1.8%), p = 0.017], respectively.

Overall, at the end of the study, compared to baseline, a decrease in the HbA1c value was observed in 86 patients, an increase was measured in 62 patients and there was no change in 5 patients. Regarding glycemic control, 13 (8.2%) patients were considered within the target for good glycemic control and 64 (41.8%) were considered to have poor glycemic control (HbA1c ≥ 9.0%). Comparisons with baseline data revealed that this difference is significant (p < 0.001, Table 2). A multivariate, stepwise and linear hierarchical analysis using the final HbA1c values (as the dependent variable) indicate that the duration of diabetes, for those that live in the city of Rio de Janeiro and have agreement between glycaemia obtained from glucometer and logbook records, were the significant independent variables. The change in *R*^*2*^ for each additional independent variable was statistically significant (p value = 0.039). The same model that used the mean HbA1c throughout the 1 year of follow-up appointments (as the dependent variable) revealed that the duration of diabetes and having agreement between glycaemia obtained from glucometer and logbook were significant independent variables. The change in *R*^*2*^ for each additional independent variable was also statistically significant (p value = 0.001).

## Discussion

This study reveals that the majority of T1D patients that were followed at a tertiary center did not have agreement between glycaemia obtained from a glucometer and their logbook records. This agreement, besides predicting the duration of diabetes, was an important predictor for having lower final levels of HbA1c and also lower mean levels of HbA1c during the 1 year follow-up. Adherence to diet was the primary factor that related to the agreement, but other factors such as the supply of strips by SUS also correlate with agreement and should be considered in clinical practice. Although the majority of our patients did not obtain good glycemic control after the 1 year follow-up appointment, most did have a significant decrease in HbA1c.

Considering the different tools for diabetes management, SMBG is one of the most important ways to obtain adequate glycemic control in patients with T1D, primarily because it helps to guide patients and providers in adjusting their insulin dose on a daily basis. Although previous studies have shown that there is an association between the frequency of SMBG and glycemic control, with a lower monitoring frequency in patients with poorer glycemic control and a negative correlation between HbA1c and SMBG [8, 12, 21–23], a frequency higher than four tests per day [21, 24] does not guarantee good metabolic control because a large proportion of patients remain out of their glycemic target, despite monitoring frequently.

In clinical practice, it is hard to evaluate the agreement of logbook records with SMBG in countries where glucometer downloads are not available during daily routines because these measures depend on the adherence of the patient to bring both the glucometer and the logbook to the clinical visit. In our study, patients that had agreement between glycaemia obtained from glucometer and logbook attained lower final HbA1c levels than patients with no agreement, having a 0.5% HbA1c reduction. Therefore, this group achieved a difference that is defined as clinically meaningful [25, 26], although not statistically significant.

Furthermore, the effectiveness of obtaining metabolic control not only depends on the agreement between the glucometer and the logbook, but also depends on the attitude towards the results found on the glucometer. During daily clinical practice, it is very difficult to find patients that adjust their treatment according to the SMBG. This can be explained by the fact that some patients and their caregivers may be afraid to change insulin doses without being advised by their physician. This is likely one of the most important barriers for achieving glycemic targets.

The majority of our patients were outside the range considered to be an appropriate glycemic control, which is a problem throughout the world [27–32]. A number of national and international audits suggest that this problem occurs even in patients attending a specialized service for intensive insulin therapy and that take part in structured education programs [7, 33]. For example, in a recent observational study that compared data from three large registries of pediatric T1D patients from England and Wales, Germany and Austria, and the USA (54.410 total children and adolescents), the overall mean HbA1c level was 8.9 ± 1.6%, 8.0 ± 1.6% and 8.3 ± 1.4%, respectively. These results reveal that these HbA1c levels are similar to those found in the group of patients that had agreement between the glycaemia measures in our study [34]. Another study that compared glycemic control among 324.501 people with T1D across all age groups through data from 19 different countries or regions throughout the world, using the same target HbA1c levels recommended by different diabetes associations, found that only 7.1% of people in the dataset had HbA1c < 6.5%, 8.7% had HbA1c of 6.5–6.9% and 12.3% had HbA1c of 7.0–7.4%. The above-mentioned data showed that the majority of individuals with T1D have higher HbA1c levels than those suggested in the guidelines [35]. This is worrisome because the results of the DCCT and the EDIC study stressed the importance of treating most patients with T1D vigorously to achieve glycemic levels as close to normal as possible, and also treating these patients as soon as possible during the course of the disease to prevent or postpone both the micro- and macro-vascular complications of this condition [3, 4].

In our study, when considering glycemic control could be separated according to HbA1c levels as good (HbA1c < 7%), intermediate (7–8.9%), poor (9.0–9.9%) and very poor (> 10%), we were able to show that people considered to have good glycemic control (at the baseline) changed to intermediate glycemic control at the end of the study. This is similar to what happened to the patients with the worst glycemic control at the baseline, who could migrate to intermediate glycemic control during the final visit. Moreover, it has already been shown that an increase in the duration of poor control will significantly reduce the likelihood of subsequent improvement [36]. A phenomenon that needs to be better characterized is called ‘glycemic tracking’ and may be responsible for the settling of the glycemic control measured using HbA1c values, onto a long-term ‘track’ that occurs on average around 5 years following the patients’ diagnosis and manifests over decades, or even a lifetime. This further increases the importance of using a targeted and aggressive metabolic control early after diagnosis [37].

Considering that it is difficult to obtain optimized glycemic control early, we can state that the complexity of T1D management is a great barrier. Besides the complexity of treatments, we should also consider other factors that influence diabetes outcomes such as parental involvement, especially for teenagers that are less psychosocially mature, partially compensating for deficits in the patient’s self-control and self-reliance. This psychological immaturity among youths may explain the reason for creating false results in their logbooks that are not in accordance with those found on the glucometer, which is a very common problem among teenagers. On the other hand, it is common for parents to impose all the responsibility for diabetes management when the patient has characteristics that indicate psychosocial maturity, which may increasingly predict poor metabolic control. In either case, it is important to emphasize the need for paternal involvement and to assess the degree of psychosocial maturity for each patient to determine future and better interventions for adolescents with poor metabolic control [38].

In developing countries, the level of literacy must be taken into account when assessing patients’ adherence to their glucose monitoring system. However, it is important to be aware that there are no instruments that are capable of detecting the main factor that influences glycemic control.

Worldwide, patients with very low economic status, which in Brazil also includes a lower level of education, are less likely to attend specialized care level clinics, which creates a negative effect on both the proportion of people with T1D that reach their glycemic control targets and on the proportion of those that have high glycemic risk. This highlights the reason that economic status must be taken into account when treating patients with T1D [39, 40]. Furthermore, we must take into account that diabetes treatment in public clinics is financed by the Brazilian National Health Care System (BNHCS), which commonly fails to provide all the health facilities and care networks that are necessary for the treatment of a patient with T1D, along with the existing regional disparities in accessing health services [41].

It is important to emphasize that adherence to diet is also a substantial tool for achieving adequate glycemic control. We found that a self-reported adherence to diet was the main factor associated with having agreement between glycaemia obtained from glucometer and the logbook records, and was also related to better glycemic control. This data is consistent with other studies that have shown an association between an adequate adherence to diet and lower levels of HbA1c [17], and that higher adherence to diet is one of the most important variables that is related to maximum adherence to the insulin therapeutic regimen, which, in turn resulted in lower mean HbA1c values [42].

Some strengths of this study should be mentioned. Patients were seen by the same medical staff during all follow-up visits at the tertiary Diabetes Unit and the entire medical team performed the data collection process through the application of a standardized and uniform questionnaire given to all the patients included in the research. Our sample included T1D patients from various ethnic groups and this study mirrors the real-life data of patients with diabetes being treated through the public medical care service in Brazil.

This study also had limitations. The number of patients evaluated and the follow-up time may have influenced our results. Furthermore, the compatibility of the time of the glucometer could not be evaluated, just as cognitive function was not assessed. Another limitation that weakens this study was not having prospectively evaluated all the included patients since their T1D diagnosis, which precludes the assessment of the glycemic tracking that is discussed above. Finally, Tanner’s stage scale was not assessed for puberty classification. However, considering the female patients in this study, the average age of menarche was 12 years, which is lower than the mean age of 12.7 years that was found in a recent nationwide survey conducted with T1D patients in Brazil [43].

Even with recent advances and improvements that include structured education programs and other self-management resources that are being used within intensive insulin therapy programs, many people with T1D continue to struggle to achieve optimal glycemic control. These new technologies and self-management schemes, which provide more flexible self-management support systems, as well as psychological counseling, not only help patients to use and choose the tools that best fit them, but mostly help them cope with their adjustments and motivation issues. Despite these advantages, not all patients consider such innovations attractive, which may be one of the reasons for the notable rate of failure for T1D patients to achieve adequate glycemic control.

## Conclusions

Our study showed that the majority of T1D patients that were followed at a tertiary center did not have significant agreement between the glycaemia obtained from a glucometer and logbook records. Adherence to diet was the main factor related to this agreement, but the supply of strips by BNHCS had a tendency to also be related to this agreement, and should be considered in clinical practice. Moreover, patients with the worst glycemic control at the baseline could migrate to intermediate glycemic control during the final visit. With regard to the secondary objectives of the study, besides the agreement between the logbook records and the glycaemia obtained from the glucometer, another important predictive factor for lower final levels and lower mean levels of HbA1c at the 1 year follow-up was the duration of diabetes.

## Data Availability

The dataset generated and analyzed during the current study is not publicly available because it contains information that may compromise research participants’ individual privacy, but is available from the corresponding author [R.C.] upon reasonable request.

## Abbreviations

T1D: Type 1 diabetes
UERJ: Rio de Janeiro’s State University
HbA1c: glycated hemoglobin
SUS: Sistema Único de Saúde
DCCT: Diabetes Control and Complications Trial
USA: United States of America
EDIC: Epidemiology of Diabetes Interventions and Complications
ADA: American Diabetes Association
CSII: continuous subcutaneous insulin infusion
SMBG: self-monitoring of blood glucose
SBD: Brazilian Diabetes Society
SED: social-educational determinants
NPH: neutral protamine hagedorn
HUPE: Pedro Ernesto University Hospital
BMI: body mass index
HPLC: high-performance liquid chromatography
SD: standard deviation
IQR: interquartile range
SPSS: Statistical Package for the Social Sciences
CIs: confidence intervals
BNHCS: Brazilian National Health Care System
